# Presentation, management, and early outcomes of young acute coronary syndrome patients- analysis of 23,560 South Asian patients from 2012 to 2021

**DOI:** 10.1186/s12872-024-04036-1

**Published:** 2024-07-19

**Authors:** Ghazal Peerwani, Bashir Hanif, Komal Abdul Rahim, Muhammad Kashif, Salim S. Virani, Sana Sheikh

**Affiliations:** 1 Department of Clinical Research Cardiology, Tabba Heart Institute, Karachi, Pakistan; 2 Department of Cardiology, Tabba Heart Institute, Karachi, Pakistan; 3 Dean’s Office, Medical College, Aga Khan University, Karachi, Pakistan; 4 Department of Community Health Sciences, Aga Khan University, Karachi, Pakistan; 5 Department of Medicine, Aga Khan University, Karachi, Pakistan; 6 Office of the Vice Provost, Research, Aga Khan University, Karachi, Pakistan

**Keywords:** Acute coronary syndrome, Young, Pakistan, In-hospital mortality, Low-middle-income country

## Abstract

**Background:**

There is dearth of literature addressing early outcomes of acute coronary syndrome (ACS) among young patients, particularly South Asians descent who are predisposed to premature coronary artery disease (CAD). Therefore, we compared presentation, management, and early outcomes of young vs. old ACS patients and explored predictors of in-hospital mortality.

**Methods:**

We extracted data of 23,560 ACS patients who presented at Tabba Heart Institute, Karachi, Pakistan, from July 2012-June 2020, from the Chest pain-MI-Registry™. We categorized data into young ≤ 45 and old ACS patients > 45 years. Chi-sq/Fischer exact tests were used to assess the difference between presentation, disease management, and in-hospital mortality between both groups. Logistic regression was used to determine odds ratio along with 95% confidence interval of factors associated with early mortality.

**Results:**

The younger patients were 12.2% and women 23.5%. The prevalence of dyslipidemia (34.5% vs. 22.4%), diabetes (52.1% vs. 27.4%), and hypertension (68.3% vs. 42.9%) was higher in older patients. Family history of premature CAD (18.1% vs. 32.7%), smoking (40.0% vs. 22.9%), and smokeless tobacco use (6.5% vs. 8.4%) were lower in older patients compared to younger ones. Younger patients were more likely to present with STEMI (33.2% vs. 45%). The median symptom-to-door time was 125 min longer (*p*-value < 0.01) in the young patients compared to the older age group. In-hospital mortality (4.3% vs. 1.7%), cardiac arrest (1.9% vs. 0.7%), cardiogenic shock (1.9% vs. 0.9%), and heart failure (1% vs. 0.6%) were more common in older patients. After adjusting for other factors, younger age (AOR 0.6, 95% CI 1.5–3.7) had significantly lesser odds of in-hospital mortality. Other factors associated with early mortality included women, family history of premature CAD, STEMI, Killip class III and IV, coronary angiography, revascularization, CABG, and use of aspirin and beta blockers within the first 24 h.

**Conclusion:**

We found every tenth ACS patient was younger than 45 years of age despite a lesser number of comorbidities such as hypertension and diabetes. Overall, the in-hospital prognosis of young patients was more favorable than that of older patients. The study emphasizes the need for tailored primary prevention programs for ACS, considering the varying risks among different age groups.

**Supplementary Information:**

The online version contains supplementary material available at 10.1186/s12872-024-04036-1.

## Background

Acute Coronary Syndrome (ACS) is a significant global health challenge, with Low-and-Middle Income countries (LMICs), particularly South Asian (SA) nations, bearing 80% of its burden and 85% of its related disability [1–4]. Recent literature has also indicated that the age-standardized ACS mortality rate was highest in lower-middle-income regions particularly in large parts of Asia compared to high-income regions which was not the case 20 years ago [5]. Not only there is heightened susceptibility in the SA population to ACS and its related adverse outcomes, but emerging evidence also suggests a demographic shift in ACS occurrence, with SAs experiencing ACS at notably younger ages (5–10 years younger) compared to other ethnic groups. This is worrisome and underscores the need for early intervention and contextual and age-appropriate healthcare approaches [6–10].

The global literature indicates that young ACS patients exhibit a distinctive clinical profile, characterized by fewer traditional risk factors like diabetes, hypertension, and dyslipidemia, but a high prevalence of a strong family history of premature coronary artery disease (CAD) and smoking habits [11–13]. Also, they present with more severe symptoms compared to their older counterparts but have better short-term outcomes [11–13]. Although there is a wealth of global literature exploring the disparity between younger and older ACS patients, SA-specific data remains very scarce especially those that cater to the entire ACS spectrum as the majority of the studies have only targeted the STEMI population [14–17].

Pakistan is a low-middle-income SA country with finite resources and an increasing burden of ACS in the younger population. Recent literature shows that the onset of ACS in Pakistan has now been pushed from 50 years to 40 and 45 years of age, particularly in men [18]. This is alarming because not only there are no ACS risk estimating tools for the younger population but also because the healthcare infrastructure of Pakistan is not equipped to cater to increasing cardiovascular crises in the younger population. In order to have tailored age-appropriate prevention and management guidelines specifically curated for Pakistan and SA countries, it is essential to have the comparison statistics between younger and older ACS patients. Therefore, we compared presentation, management, and in-hospital outcomes of young vs. old ACS patients and explored predictors of in-hospital mortality in Pakistan.

## Methodology

### Study design and setting

We extracted data from 23,560 ACS patients presented at Tabba Heart Institute (THI), Karachi, Pakistan, from July 2012 to June 2020, from the Chest pain-MI Registry™. THI is a not-for-profit, tertiary-level, cardiac-only hospital in Karachi, Pakistan. The site serves as a key referral site for cardiovascular emergencies and receives 4000 ACS cases annually from all over Pakistan on average. The hospital is among the few hospitals in Pakistan that are paper-free and maintain all patients’ records in an electronic medical record (EMR) system. THI has been affiliated with the National Cardiovascular Data Registry (NCDR^®^) since 2013 and received the “Platinum Performance Achievement Award” for the Chest Pain-MI™ registry in 2022 and 2023 for its compliance with American College of Cardiology guidelines for treating myocardial infarction (MI).

### Participants

All the patients presenting to THI during the study period and diagnosed with ACS that is ST Elevation Myocardial Infarction (STEMI), Non-ST Elevation Myocardial Infarction (NSTEMI), and unstable angina, were included at admission. The patients were divided into two groups, i.e., young ACS ≤ 45 years of age patients and older ACS more than 45 years of age. No exclusion criteria were applied.

### Variables of interest

The clinical co-variates such as diabetes, hypertension, and dyslipidemia are recorded in the database as reported by physicians in electronic medical records (EMR). Physicians diagnosed hypertension as the presence of at least one minimum systolic blood pressure of 140 mmHg, or minimum diastolic blood pressure of 90 mmHg, or a history of taking antihypertensive drugs [19]. Dyslipidemia was labeled by physicians if total cholesterol at the time of presentation was ≥ 200 mg/dL, or or low-density lipoprotein cholesterol levels of ≥ 116 mg/dL, or if the home medications of patients included any lipid-lowering drugs [20]. Diabetes mellitus type 2 was defined as fasting blood sugar ≥ 126 mg/dL, or HbA1c of ≥ 6.5%, or a history of taking anti-diabetic medications. Information about ACS disease management and clinical outcomes was reported by physicians and extracted from EMR for entry into the database. The patient demographics, family history, and tobacco consumption were self-reported by patients when the registry team interviewed them at the time of admission.

The study’s primary exposure was age at ACS, and the study outcome was in-hospital mortality i.e., death of patients during the index admission. Mortality while presenting with symptoms in the emergency or during the hospital stay in the cardiac care unit or wards was taken as in-hospital mortality. Other outcomes included any complications that occurred during the same hospital stay. The complications are defined as per NCDR^®^ Chest pain-MI Registry™ [21] and include myocardial infarction, cardiogenic shock, heart failure, cerebrovascular accident (CVA)/stroke, a new requirement for dialysis, hematoma at the access site, and cardiac arrest.

### Statistical analysis

All the analysis was done using STATA version 15.0. Descriptive data analysis included estimating summary measures for continuous variables by mean and standard deviation (SD) such as left ventricle ejection fraction, or median and interquartile range (IQR) such as symptom-to-door time. Categorical variables were presented as frequencies and percentages such as gender, payor status, etc. The baseline characteristics, disease presentation, management, and outcomes of Young ACS cases were compared using chi-square and independent students’ t-tests with old ACS cases. If assumptions of normality were not met or data was sparse, alternate non-parametric tests like Mann-Whitney, fisher exact, or linear-by-linear association were used to compare the groups. Multivariable logistic regression was applied to determine factors associated with in-hospital mortality between the two groups, and crude and adjusted odds ratio (AOR) with 95% confidence interval (CI) were estimated. The in-hospital mortality model was adjusted for age, gender, type of ACS, Killip class, prior history of ACS, revascularization, and medications including aspirin and beta-blocker received within 24 h of the same admission. A *p*-value of < 0.05 was considered statistically significant.

## Results

### Sociodemographic characteristics, medical history, and presentation

The mean age was 59.0 ± 11.4 years. The young patients were 12.2% of the sample, and the women were 14.2% in the young ACS group compared to 24.8% in the older group (*p*-value < 0.01). Table 1 shows that there was a significant difference between the risk factors such as hypertension, diabetes, and dyslipidemia between the ≤ 45 and > 45 years age group, with higher proportions in the older age group (*p*-value < 0.01). Tobacco smoking was less prevalent in the older age group (6.5% vs. 8.4%; *p*-value 0.02) compared to the younger group. Previous history of coronary artery disease (CAD), heart failure, and revascularization were higher in the > 45 years age group, whereas family history of premature coronary artery disease (CAD) was less likely in older age group (18% vs. 32.7%). The most common presentation in the ≤ 45 years group was STEMI, and it was NSTEMI in the > 45 years group. Single vessel disease was prevalent in the younger group (49.8%) whereas triple vessel disease was found in 31.1% of the older group. The median symptom-to-door time was 125 min longer (*p*-value < 0.01) in the young ACS patients compared to the other age group However, for those who underwent PCI, door to balloon time and symptom to balloon time was not different between the groups(*p* > 0.05) (Table 1). Baseline medications of the patients that they were taking at home before admission are given in supplemental Table 1.

**Table 1:** Comparison of baseline characteristics of ACS patients ≤ 45 years of age and > 45 years of age (*n* = 23,560)

Variables	Total *n* (%) *n* = 23,560	Young ≤ 45 years *n* (%) *n* = 2,894	> 45 years *n* (%) *n* = 20,666	*p*-value
Sociodemographic				
Women	5547 (23.5)	412 (14.2)	5135 (24.8)	< 0.01**
Paying status: Welfare	721 (3.1)	111 (3.8)	610 (3.0)	0.01*
Dyslipidemia	7782 (33.0)	648 (22.4)	7134 (34.5)	< 0.01**
Hypertension	15,357 (65.2)	1242 (42.9)	14,115 (68.3)	< 0.01**
Diabetes mellitus	11,565 (49.1)	794 (27.4)	10,771 (52.1)	< 0.01**
Tobacco use:				
Smoking Smokeless	5693 (24.2) 603 (6.7)	1172 (40.0) 82 (8.4)	4735 (22.9) 521 (6.5)	< 0.01** 0.02*
Family history of premature CAD	4680 (19.9)	947 (32.7)	3733 (18.1)	< 0.01**
Prior MI	4086 (17.3)	348 (12.0)	3738 (18.1)	< 0.01**
Prior heart failure	796 (3.4)	31 (1.1)	765 (3.7)	< 0.01**
Prior PCI	2851 (12.1)	173 (6.0)	2678 (13.0)	< 0.01**
Prior CABG	1545 (6.6)	37 (1.3)	1508 (7.3)	< 0.01**
Prior CAD	5636 (24.0)	362 (12.5)	5274 (25.6)	< 0.01**
Disease type:				
NSTEMI STEMI Unstable angina	11,339 (48.1) 8173 (34.7) 4048 (17.2)	1170 (40.4) 1302 (45.0) 422 (14.6)	10,169 (49.2) 6871 (33.2) 3626 (17.5)	< 0.01**
Number of diseased vessels:				
Single Double Triple	8695 (36.9) 5721 (24.2) 6835 (29.0)	1441 (49.8) 596 (20.6) 408 (14.1)	7254 (35.1) 5125 (24.8) 6427 (31.1)	< 0.01**
Signs and symptoms at presentation				
Killip class III Killip class IV	2505 (10.6) 579 (2.5)	107 (3.7) 52 (1.8)	2398 (11.6) 527 (2.6)	< 0.01**
LVEF (%) ^	48.3 ± 11.4	48.2 ± 12.1	48.3 ± 11.3	0.89
Cath	18,007 (95.1)	2286 (94.0)	15,721 (95.3)	< 0.01**
PCI	9983 (52.7)	1498 (61.6)	8485 (51.4)	< 0.01**
CABG	2725 (11.6)	173 (6.0)	2552 (12.4)	< 0.01**
Median symptom onset to door time^^	649 (187, 2788)	760 (192, 3140)	635 (186, 2736)	< 0.01**
Median door to balloon time^a^	70 (55, 95)	67 (54, 88)	70 (55, 95)	0.10
Median symptom to balloon time^a^	265 (172, 470)	255 (163, 455)	265 (175, 470)	0.11

ACS = Acute coronary syndrome; CABG = coronary artery bypass grafting; CAD = coronary artery disease; LVEF = left ventricular ejection fraction; MI = myocardial infraction; NSTEMI = non-ST elevation myocardial infraction; PCI = percutaneous coronary intervention; STEMI = ST-elevation myocardial infraction

**p-value < 0.01 (highly significant); *p-value < 0.05 (significant)

^Student’s t-test of independent sample; ^^Mann Whitney test

^a^ data is available only for those who had PCI; data of 6293 out 9983 PCI cases is missing

### Medications within 24 h and at discharge

No significant difference was found in the medication prescriptions within 24 h of admission and at the time of discharge between the two age groups except angiotensin-converting enzyme/angiotensin receptor blocker (ACE/ARB) inhibitors at discharge, aspirin and beta-blockers within 24 h of admission. These medications were prescribed less in > 45 age group compared to their younger counterparts (*p*-value < 0.01) (Table 2).

**Table 2:** Comparison of medications given within 24 h of admission and at discharge between younger and older ACS patients

Variables	Total *n* (%) *n* = 23,560	Young ≤ 45 years *n* (%) *n* = 2,894	> 45 years *n* (%) *n* = 20,666	*p*-value
ACE inhibitors/ARB within 24 h	7308 (31.0)	934 (32.3)	6374 (30.9)	0.11
ACE inhibitors/ARB prescribed at discharge	15,221 (64.6)	2042 (70.6)	1317 (63.9)	< 0.01**
Aspirin within 24 h	22,275 (94.5)	2769 (95.7)	19,506 (94.5)	< 0.01**
Aspirin was prescribed at discharge	21,033 (89.3)	2584 (89.3)	18,407 (89.3)	0.92
Statins within 24 h	19,042 (80.8)	2345 (81.1)	16,697 (80.8)	0.73
Statins prescribed at discharge	21,016 (89.2)	2587 (89.4)	18,492 (89.3)	0.83
Beta-blockers within 24 h	13,280 (56.4)	1735 (60.0)	11,545 (55.9)	< 0.01**
Beta-blockers prescribed at discharge	19,762 (83.9)	2445 (84.5)	17,317 (83.9)	0.43
Anticoagulants administered during hospitalization	21,837 (92.7)	2684 (92.7)	19,153 (92.7)	0.93

ACE/ARB = angiotensin-converting enzyme/angiotensin receptor blocker

**p-value < 0.01 (highly significant)

Chi^2^ test of association applied

### In-hospital mortality and complications

The overall in-hospital mortality was 4.0%, significantly higher in the > 45 years age group compared to the ≤ 45 years group (4.3% vs. 1.7%), as shown in Table 3. Complications including cardiogenic shock, heart failure, new requirement for dialysis, and cardiac arrest were also significantly higher in the older group (*p*-value < 0.01) **(**Table 3**).**

**Table 3:** Comparison of in-hospital mortality and complications between younger and older ACS patients

Variables	Total *n* (%) *n* = 23,560	Young ≤ 45 years *n* (%) *n* = 2894	> 45 years *n* (%) *n* = 20,666	*p*-value
In-hospital mortality	945 (4.0)	49 (1.7)	896 (4.3)	< 0.01**
Any complication	1391 (5.9)	96 (3.3)	1295 (6.3)	< 0.01**
Myocardial infarction	66 (0.3)	7 (0.2)	59 (0.3)	0.67
Cardiogenic shock	423 (1.8)	26 (0.9)	397 (1.9)	< 0.01**
Heart failure	226 (1.0)	18 (0.6)	208 (1.0)	0.05
CVA/stroke	38 (0.2)	4 (0.1)	34 (0.2)	1.0
New requirement for dialysis	111 (0.5)	2 (0.1)	109(0.5)	< 0.01**
Hematoma at the access site	136 (0.6)	14(0.5)	122(0.6)	0.47
Cardiac arrest	402 (1.7)	20 (0.7)	382 (1.8)	< 0.01**

ACS = acute coronary syndrome; CVA = cerebrovascular accident

**p-value < 0.01 (highly significant)

Chi^2^ test and Fischer exact test of association applied

### Factors associated with in-hospital mortality

Table 4 shows the multivariable model of factors associated with in-hospital mortality. The in-hospital mortality was 40% less likely in the younger group compared to the older group after adjusting for gender, comorbidities, presenting signs and symptoms, and disease management (AOR: 0.6, (0.4,0.9)). Higher odds of in-hospital mortality were seen in women, patients with a history of heart failure, STEMI at presentation, multi-vessel disease, Killip class III/IV at presentation, procedures of coronary angiography, revascularization, and CABG. In patients with a history of diabetes mellitus, a family history of premature CAD, and given aspirin and beta-blocker during the first 24 h of admission, the in-hospital mortality was lower.

**Table 4:** Factors associated with in-hospital mortality in ACS patients

Variables	Crude odds ratio (95% CI)	Adjusted odds ratio (95% CI)
Age Category (Ref: >45 years) ≤ 45 years	0.38 (0.28, 0.50)	0.6 (0.4, 0.9) *
Gender (Ref: Men)		
Women	1.3 (1.1, 1.5)	1.3 (1.0, 1.5)
Paying status (Ref: Self-paid)	0.78 (0.56, 1.1)	-
Welfare		
Dyslipidemia (Ref: no dyslipidemia)	0.83 (0.72, 0.96)	0.9 (0.7, 1.1)
Hypertension (Ref: no hypertension)	1.1 (1.0, 1.3)	1.1 (0.9, 1.3)
Diabetes mellitus (Ref: no diabetes mellitus)	1.4 (1.2, 1.6)	1.2 (1.0, 1.5)
Family history of premature CAD (Ref: no family history)	0.48 (0.39, 0.59)	0.6 (0.4, 0.8) *
Prior MI (Ref: no prior MI)	1.0 (0.8, 1.2)	-
Prior heart failure (Ref: no prior heart failure)	2.3 (1.8, 3.0)	1.2 (0.8, 2.0)
Prior PCI (Ref: no prior PCI)	0.75 (0.60, 0.94)	0.8 (0.6, 1.1)
Prior CABG (Ref: no prior CABG)	1.0 (0.76, 1.3)	-
Prior CAD (Ref: no prior CAD)	1.0 (0.83, 1.1)	-
Disease type (Ref: unstable angina)		
NSTEMI STEMI	2.0 (1.5, 2.6) 5.5 (4.2, 7.3)	1.4 (0.8, 2.2) * 5.9 (3.7, 9.3) *
Killip class III-IV (Ref: Killip class I & II)	9.0 (7.9, 10.3)	6.5 (5.5, 7.7) *
LVEF (%)	0.89 (0.85, 0.93)	
Number of diseased vessels:		
Single Double Triple	2.3 (1.5, 8.3) 3.7 (2.3, 5.9) 5.3 (3.4, 8.5)	1.6 (0.9, 2.6) 2.1 (1.3, 3.5) 2.7 (1.6, 4.4)
Cath (Ref: no Cath)	2.2 (1.3, 3.7)	1.9 (1.1, 3.3) *
PCI (Ref: no PCI)	1.3 (1.1, 1.5)	1.4 (1.1, 1.7) *
CABG (Ref: no CABG)	1.5 (1.2, 1.8)	2.2 (1.7, 2.8) *
Aspirin within 24 h (Ref: no aspirin within 24 h)	0.73 (0.56, 0.94)	0.6 (0.4, 0.8) *
Beta-blockers within 24 h (Ref: no beta-blockers within 24 h)	0.31 (0.26, 0.35	0.4 (0.3, 0.5) *

CABG = coronary artery bypass grafting; CAD = coronary artery disease; LVEF = left ventricular ejection fraction; MI- myocardial infraction; NSTEMI = non-ST elevation myocardial infraction PCI = percutaneous coronary intervention; STEMI = ST elevation myocardial infraction

Simple and Multiple Logistic Regression applied.

implies to variables insignificant at univariate analysis (*p* > 0.25)

*Variables significant in multivariable analysis

## Discussion

The study found that 12.2% of the sample was younger (≤ 45 years of age) and had a lower prevalence of traditional CVD risk factors compared to the > 45 years age group, except for smoking and a family history of premature CAD, which were higher in the younger group. STEMI was the most common clinical presentation of ACS in the younger group. The median symptom onset to door time was higher in younger patients. The younger group had a 40% lower adjusted mortality risk than the > 45 age group. No difference in adjusted odds of mortality was found between men and women. Presenting with STEMI and undergoing diagnostic catheterization and revascularization were associated with higher odds of early mortality.

We defined young patients as ≤ 45 years of age; however, in the literature from South Asia, the age cut-off to categorize younger age groups has varied from < 40 [22], ≤ 50 [23] to < 54 [24] years of age. Hence, comparing the proportion of younger cases in our study with the other studies is difficult. It is observed that the proportion of younger cases reduces with the lowering of the age cut-off. Ullah et al. reported 52.6% of young cases at < 54 years of age [24], whereas Deora et al. found 10% cases at the cut-off of < 40 years of age [22]. In our study, 12.2% of the ACS cases were ≤ 45. In our study, young patients constituted 12.2% of total ACS cases, which aligns with the findings of another study conducted in India (10.4%) using same-age cutoffs [25]. Conversely, a European study has reported a much lower proportion of young ACS patients having similar cutoffs (7.9%) [26]. These differences can be attributed to racial and sociodemographic differences. Literature has emphasized the influence of socioeconomic factors including poverty, housing, awareness, income, access to nutrition as well as occupational stresses to play a vast role in the occurrence and outcomes of ACS [27–29]. Pakistan is an LMIC with resource limiting settings as well as a higher poverty rate, these factors might have played a role in a higher proportion of young population getting ACS. One study also quoted that SAs had poor health behaviors compared to other ethnicities such as lack of moderate to high-intensity exercises and low intake of fruits and vegetables making them more prone to cardiovascular disorders early in life [9]. Additionally, the role of genetics in SAs cannot be overlooked, as genetic factors may also contribute significantly to increased predisposition to early incidence of ACS. Further studies are warranted to explore the effect of sociodemographic factors as well as genetics on the early incidence of ACS in the SA population. The young ACS cases comprised 85.8% of men. A higher proportion of men (more than 80%) in young ACS has also been found in other studies [30, 31]. Our results are consistent with the literature that a family history of premature CAD was frequent in the young group compared to the other group. Other modifiable risk factors such as hypertension, diabetes, and dyslipidemia are less prevalent in the younger group compared to the older group [24, 32. Existing literature and results of our study suggest that a family history of premature CAD could be a detrimental, non-modifiable factor in causing the disease at a younger age. Leander et al. found a synergistic effect of smoking and a family history of premature CAD to cause MI in their Stockholm Heart Epidemiology Program (SHEEP) [33]. The evidence emphasizes the need to strengthen the interventions to reduce tobacco use to prevent CAD in the young population.

STEMI was the most common presentation of ACS in the young group in our study. The findings are consistent with the literature that reported about 3/4th of STEMI in the young group compared to older patients [26, 34]. The pathophysiology of a higher number of STEMI cases in younger patients is mainly due to plaque morphology (less fibrotic content), resulting in unstable plaques prone to plaque rupture [35]. Young age and smoking were found to be associated with unstable plaque formation [35]. Our study and other investigators found a higher number of tobacco users among young patients compared to older ones [30]. Schoeneberger et al. also reported mild to moderate stenosis among young patients may make them more vulnerable to STEMI compared to old patients with moderate to severe stenosis due to the presence of collateral circulation among older adults presenting with ACS [34].

In our study, we observed that a higher proportion of young patients received Aspirin and beta blockers within the first 24 h of admission compared to older patients. A plausible explanation for this trend is that a higher proportion of young patients presented with ST-Elevation Myocardial Infarction (STEMI), a life-threatening condition characterized by more prominent symptoms, hence they were administered early compared to their older counterparts. Despite the significant difference, it is important to note that the variation is not substantial from a statistical standpoint. Also, no differences are seen in prescription of other drugs in first 24 h between younger and older patients. Our practices follow a standard protocol which is in compliance with American College of Cardiology Guidelines for treating MI [36] and therefore in terms of administration of immediate medications, no age-based discrimination is done ensuring that all patients, regardless of age, receive optimal and timely care based on established clinical guidelines.

Our study findings indicated higher median symptom onset to door timing in younger populations compared to older ones. Rafi et al. also reported similar findings with the younger population (< 40 years) having 2.43 times higher odds of having hospital delay compared to patients > 60 years of age [37]. It is usually a conventional belief that ACS is the disease of old and that is the reason many younger patients attribute their symptoms to other conditions including heartburn etc. causing a delay in first medical contact [37]. Additionally, previous history of MI or heart failure or previous CABG or revascularization might be the reason older patients might be more vigilant and proactive in seeking immediate medical care. However, the literature is very inconsistent with this finding as several studies suggest lesser median symptom onset to door time to first medical contact in younger patients compared to older ones [38–40]. Probable reasons as quoted by studies suggest that younger patients are more likely to present with STEMI and, therefore have more intense symptoms of typical ischemia which may prompt swifter medical attention [41]. Conversely, older patients often presenting with NSTEMI, might exhibit altered pain thresholds, atypical symptoms as well as multiple other comorbidities due to which there might be a delayed symptom onset to door time [42]. Further studies are warranted to explore this inconsistency.

We found higher compliance with the American College of Cardiology guidelines for discharge medications post-MI for both young and old patients. It must be emphasized that the study is single-center and may not reflect the general compliance of the country’s clinicians with the guidelines. Our study found significantly lower odds of in-hospital mortality in younger cases of ACS than in older cases. Ricci et al. presented similar results where the incidence of 30-day mortality was 1.3% in younger patients and 6.9% in older patients [43]. Another study from Saudia Arabia documents only one death due to ACS of the 924 patients; the death was observed in older age patients [44]. This may be attributed to fewer comorbidities, faster recovery rates, and limited atherosclerosis progression [45]. However, individual variations, timely access to medical care, and ongoing management adherence remain crucial in determining the prognosis of ACS in younger populations [46].

PCI is widely regarded as a highly efficacious and safe intervention for ACS management, with its advantages typically surpassing potential risks [47]. However, in our study patients who underwent PCI were found to be at higher odds of in-hospital mortality. The existing literature presents a contradiction to this finding., Multiple studies report improved clinical outcomes and lesser mortality in patients who underwent primary PCI [48, 49]. The findings in our study can have several possible explanations. Early mortality could be attributed to the critical condition of patients upon admission, indicating a poor prognosis. Additionally, the literature suggests that patients with affected left dominant anatomy who underwent PCI had higher in-hospital mortality which might also be our case [50]. Delay in time from first medical contact to PCI can also be an alternate explanation that may impact patient outcomes [51]. As our findings contradict the present literature, we suggest further studies to explore early mortality in the entire spectrum of ACS patients. We also warrant readers to interpret the findings considering our limitation to investigate the mentioned pathophysiology and several other post-procedural factors, lifestyle, and genetic confounders that might influence this association.

### Strengths and limitations

The key strengths of the study are that it encompasses the whole ACS spectrum and a large volume of 20,000 patients. There have been multiple studies on premature atherosclerosis from the region; however, most of the literature is focused on STEMI patients.

The results of the study should be interpreted considering its limitations. The first limitation is that the study is a single-center study, and though the center receives patients from all sociodemographic strata, the generalizability should still be carefully evaluated. The study hospital strictly follows the American College of Cardiology guidelines for patient management; hence, the patient’s outcomes could differ from those of other centers in the country.

## Conclusion

One out of ten patients of ACS was less than 45 years of age. The main drivers of ACS in the younger age group were a family history of premature CAD and smoking, whereas in the older age group, diabetes, hypertension, and dyslipidemia were prevalent. A higher proportion of younger patients were men and presenting with STEMI. Overall, the younger group had a favorable prognosis compared to the older group. The study reflects the need for tailored programs for primary prevention of ACS based on the prevalent risks in different age groups.

### Electronic supplementary material

Below is the link to the electronic supplementary material.

Supplementary Material 1

## Data Availability

The data that support the findings of this study will be available upon the request to the corresponding author. Restrictions apply to the availability of the data as it is not publicly available.

## Abbreviations

ACS: Acute Coronary Syndrome
AOR: Adjusted odds ratio
CAD: Coronary Artery Disease
CABG: Coronary Artery Bypass Grafting
CI: Confidence Interval
CVA: Cerebrovascular Accident
EMR: Electronic Medical Record
HIC: High income country
LMIC: Low-and-Middle Income Country
LVEF: Left Ventricular Ejection Fraction
MI: Myocardial Infarction
NCDR: National Cardiovascular Data Registry
OR: Odds Ratio
PCI: Percutaneous Coronary Intervention
SA: South Asian
STEMI: ST Elevation Myocardial Infarction
NSTEMI: Non-ST Elevation Myocardial Infarction
SD: Standard Deviation
THI: Tabba Heart Institute
