# Supplementary material for: Presentation, management, and early outcomes of young acute coronary syndrome patients- analysis of 23,560 South Asian patients from 2012 to 2021
Source: BMC Cardiovasc Disord. 2024 Jul 19;24:378. doi: 10.1186/s12872-024-04036-1 (PMC11264989; doi:10.1186/s12872-024-04036-1)
Supplement: Supplementary file 1 — Supplementary Material 1 [file 12872_2024_4036_MOESM1_ESM.docx]

**Supplemental Table 1: Number of patients on home medications for hypertension, diabetes, and dyslipidemia before the ACS event.**

| **Type of antihypertensive*** | **Total Hypertensives**  **n (%)**  **n=15357** | **Hypertensives ≤45 years**  **n (%)**  **n=1242** | **Hypertensives >45 years**  **n (%)**  **n=14115** |
| --- | --- | --- | --- |
| ACE inhibitors | 2272 (14.7) | 183 (14.7) | 2089 (14.8) |
| Beta blockers | 6055 (39.4) | 395 (31.8) | 5660 (40.1) |
| ARB | 3390 (22.1) | 200 (16.1) | 3190 (22.6) |
| **Type of diabetes therapy** | **Total Diabetics**  **n (%)**  **n=11565** | **Diabetics ≤45 years**  **n (%)**  **n=794** | **Diabetics >45 years**  **n (%)**  **n=10771** |
| Diet | 434 (3.7) | 35 (4.4) | 399 (3.7) |
| Oral | 7553 (65.3) | 552 (69.5) | 7001 (65.0) |
| Insulin | 2774 (23.9) | 145 (18.2) | 2629 (24.4) |
| **Treatment for dyslipidemia** | **Total Dyslipidemics**  **n (%)**  **n=7782** | **Dyslipidemics ≤45 years**  **n (%)**  **n=648** | **Dyslipidemics >45 years**  **n (%)**  **n=7134** |
| Statins | 5023 (64.5) | 358 (55.2) | 4665 (65.4) |

*The numbers are not mutually exclusive. One patient can be on more than one medication.
